# Novel Approaches in Cardiac Imaging for Non-invasive Assessment of Left Heart Myocardial Fibrosis

**DOI:** 10.3389/fcvm.2021.614235

**Published:** 2021-04-15

**Authors:** Giulia Elena Mandoli, Flavio D'Ascenzi, Giulia Vinco, Giovanni Benfari, Fabrizio Ricci, Marta Focardi, Luna Cavigli, Maria Concetta Pastore, Nicolò Sisti, Oreste De Vivo, Ciro Santoro, Sergio Mondillo, Matteo Cameli

**Affiliations:** ^1^Department of Medical Biotechnologies, Division of Cardiology, University of Siena, Siena, Italy; ^2^Section of Cardiology, Department of Medicine, University of Verona, Verona, Italy; ^3^Department of Neuroscience, Imaging and Clinical Sciences, Institute of Advanced Biomedical Technologies, “G.d'Annunzio” University of Chieti-Pescara, Chieti, Italy; ^4^Department of Clinical Sciences, Lund University, Malmö, Sweden; ^5^Casa di Cura Villa Serena, Città Sant'Angelo, Italy; ^6^Department of Advanced Biomedical Science, Federico II University Hospital Naples, Naples, Italy

**Keywords:** fibrosis, echocardiography, myocardial strain, speckle tracking, cardiac magnetic resonance

## Abstract

In the past, the identification of myocardial fibrosis was only possible through invasive histologic assessment. Although endomyocardial biopsy remains the gold standard, recent advances in cardiac imaging techniques have enabled non-invasive tissue characterization of the myocardium, which has also provided valuable insights into specific disease processes. The diagnostic accuracy, incremental yield and prognostic value of speckle tracking echocardiography, late gadolinium enhancement and parametric mapping modules by cardiac magnetic resonance and cardiac computed tomography have been validated against tissue samples and tested in broad patient populations, overall providing relevant clinical information to the cardiologist. This review describes the patterns of left ventricular and left atrial fibrosis, and their characterization by advanced echocardiography, cardiac magnetic resonance and cardiac computed tomography, allowing for clinical applications in sudden cardiac death and management of atrial fibrillation.

## Introduction

**Figure 1:**
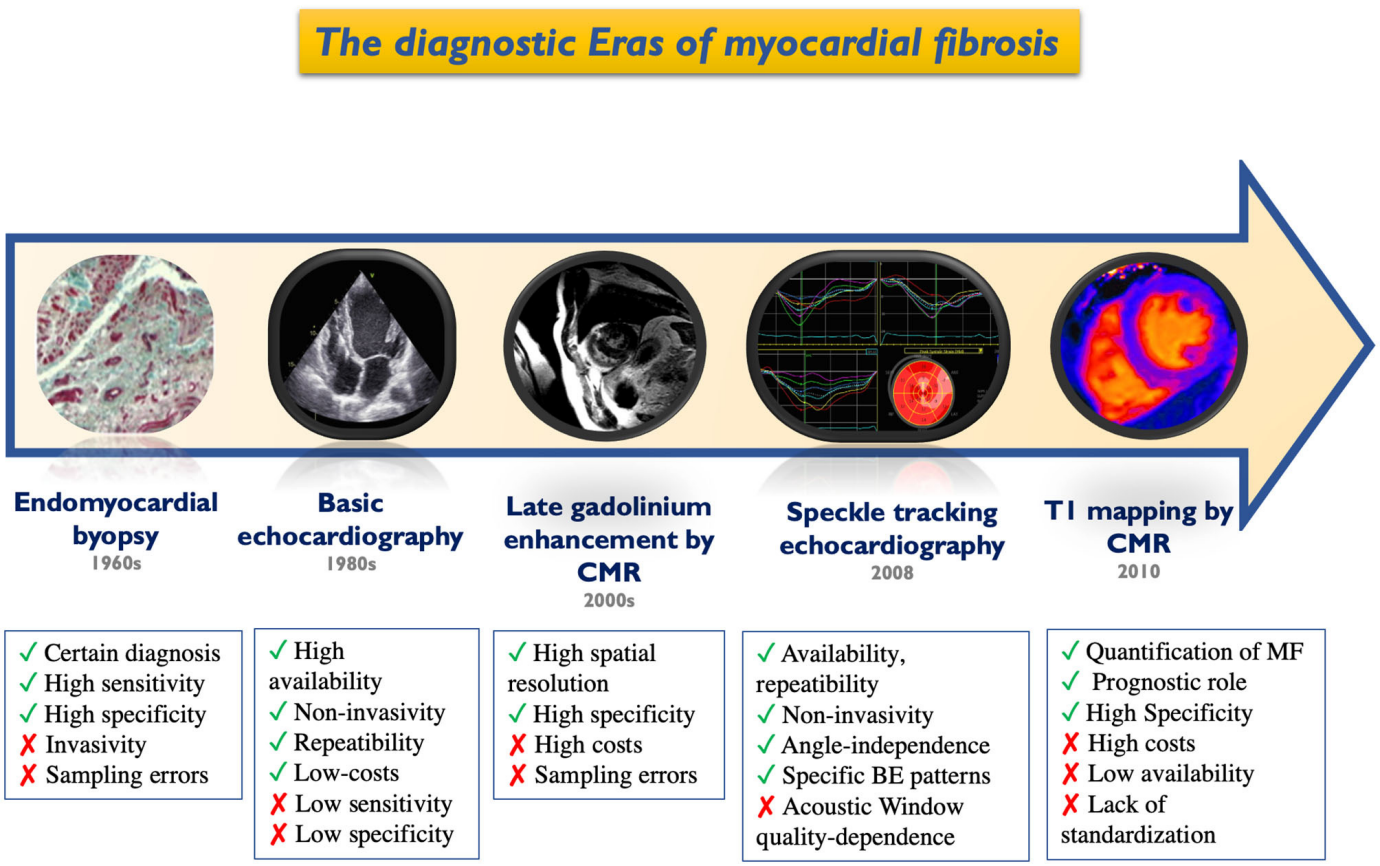
Historical timeline of cardiac imaging evolution for the detection of myocardial fibrosis. BE, bull-eye; CMR, cardiac magnetic resonance; MF, myocardial fibrosis.

Myocardial fibrosis (MF) has become a crucial marker to identify on multi-modality imaging. Advanced echocardiographic techniques, such as myocardial deformation indices by speckle tracking echocardiography (STE), allow objective identification of abnormalities in cardiac function in early and subclinical phases of various cardiac diseases (1). Over the last decade, cardiovascular magnetic resonance (CMR) has emerged as a powerful non-invasive imaging modality able to characterize the myocardial tissue. Late gadolinium enhancement (LGE) imaging is a fully-established technique for non-invasive replacement MF detection, whereas parametric T1-mapping indices yield high diagnostic accuracy for the detection of diffuse interstitial MF (2). Cardiac computed tomography (CT) with iodine contrast has also been revealed as a possible integrative imaging technique for detecting left ventricular myocardial abnormalities. The potential of these novel imaging techniques for myocardial tissue characterization has increased interest in the investigation of MF, by providing additional data for translational and clinical research. Figure 1 shows the evolution, starting from invasive endomyocardial biopsy (EMB), of cardiac imaging techniques for the detection of MF, with their main pros and cons. This review focuses on detecting MF by cardiac imaging and its current clinical and prognostic significance, only describing left heart chambers where significant evidence is currently available in the literature.

## Left Ventricular Fibrosis

### Mechanisms and Patterns of Left Ventricular Fibrosis

**Figure 2:**
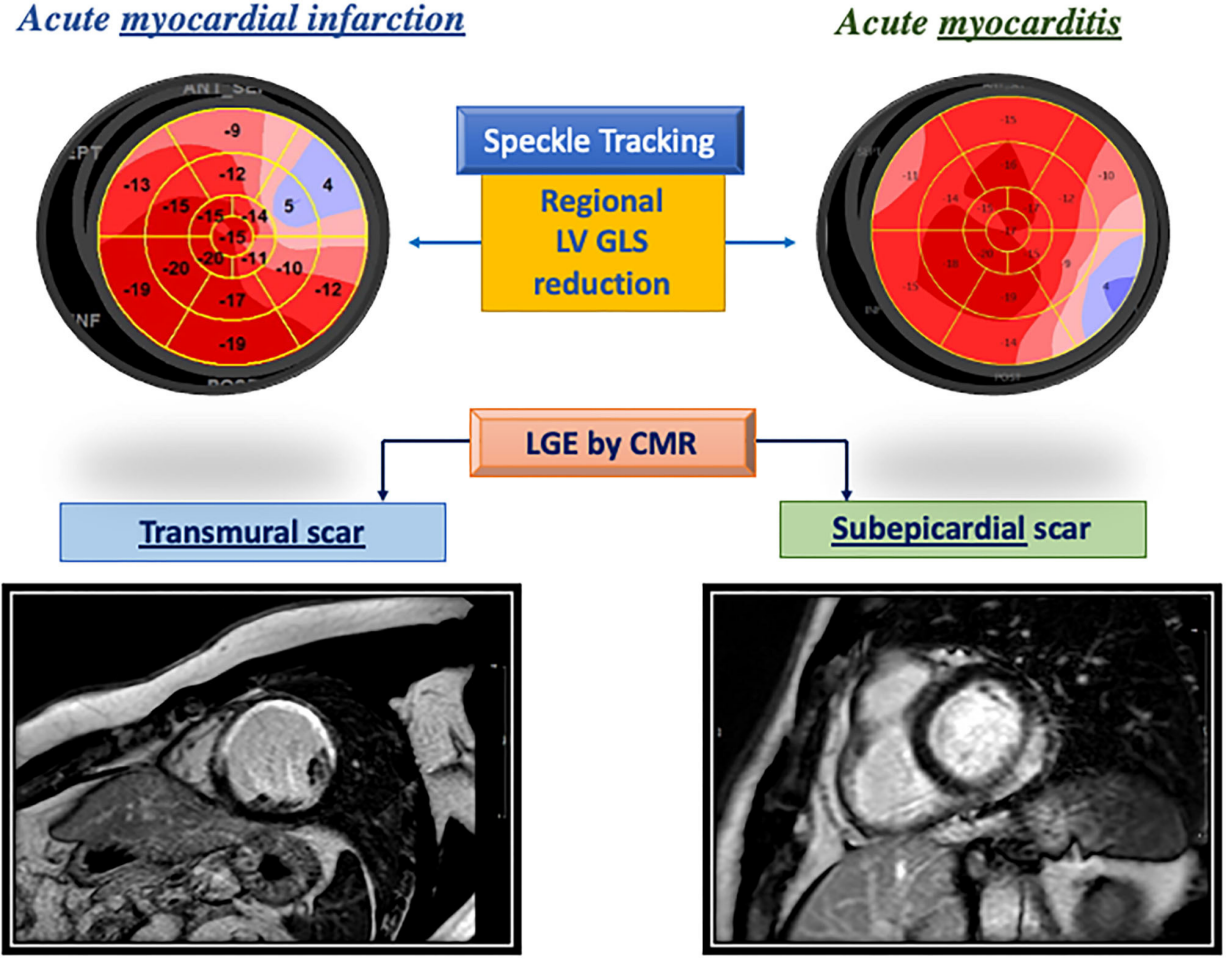
Different patterns of left ventricular fibrosis in an acute myocardial infarction (left) and in acute myocarditis (right) as highlighted by impairment in segmental global longitudinal strain reduction at bull's eye and the corresponding region of late gadolinium enhancement.

In the presence of MF, the excessive activation of cardiac fibroblasts (CFs) is responsible for the progressive expansion of the extracellular matrix (ECM) to the detriment of cardiomyocytes (3). ECM surrounds myocytes and vasculature cells as a scaffold and controls biochemical signals. In response to different types of injuries, the activation of inflammatory cells and cytokines causes a marked proliferation of CFs and collagen production and other ECM proteins (4). MF starts as an adaptive process, but progressively leads to the distortion of myocardial architecture and the loss of contractile function. MF can be primary, due to a primitive myocardial involvement both for genetic or non-genetic causes (including dilated, hypertrophic, and arrhythmogenic cardiomyopathies) or secondary to myocardial damage as seen in myocarditis, valvular heart disease or myocardial infarction (Table 1). Primary or secondary myocardial injury may lead to an adverse myocardium interstitial remodeling, that represents a landmark of different pathophysiological paths, all characterized by an ECM excessive deposition of proteins by myofibroblasts (4). In the past, invasive studies, including anatomopathological samples analysis, helped to correlate the different cardiac diseases to a peculiar fibrotic pattern. When fibrosis is reactive, LV remodeling features collagen overproduction with consequent expansion of the interstitial compartment in response to triggers such as increased pre-/post-load of the LV. This category includes valvular heart disease, hypertension or chronic kidney disease, where the increased wall stress induces pro-fibrogenic cytokines release. Coronary artery disease leads to an inadequate oxygen supply to cardiomyocytes, with consequent atrophic and necrotic changes, progressing to replacement fibrosis. Post-ischemic fibrosis may consist of isolated well-demarcated scars or interspersed scars surrounding normal contractile cells (“tiger spotted” aspect). Similar pathogenesis can be observed in myocarditis, where the etiology is primarily inflammatory. Figure 2 shows differences between the LV transmural scar caused by acute myocardial infarction and the epicardial scar characterizing acute myocarditis, detected with STE and LGE CMR. The peculiar fibro-fatty infiltration in ventricular arrhythmogenic heart disease would derive from the precarious desmosome integrity and cell loss consequent to gene mutations involving desmoglein, desmocollin or desmoplakin. A peculiar ECM expansion can be found in infiltrative diseases like amyloidosis, resulting from the deposition of insoluble fibrils. In this scenario, organ dysfunction conveys a hypertrophic-restrictive phenotype due to tissue disorganization induced by amyloid deposits with downstream reactive fibrosis.

**Table 1:** Main causes of left ventricular primary and secondary myocardial fibrosis and typical features on late gadolinium enhancement cardiac magnetic resonance.

**Primary myocardial fibrosis**		
**Causes**	**Clinical entity**	**LGE features**
**Genetic**		
	Hypertrophic cardiomyopathy	- Patchy non-ischemic pattern LGE, particularly in those walls with the greatest hypertrophy - LGE at the RV septal insertion sites (not specific)
	Fabry disease	- LGE is related to the extent of LV hypertrophy - Mid-wall or subepicardial LGE located in the basal inferolateral wall
	Idiopathic Dilated Cardiomyopathy	- “Mid-wall” stripe: intramural LGE usually in the basal and/or mid septum. - Some cases patchy or diffuse striated LGE - Not related to a particular coronary arterial territory
**Non-genetic**		
	Amyloidosis	- Focal fibrosis with circumferential subendocardial LGE most pronounced at the base and middle of the ventricle - Diffuse subendocardial LGE - Difficult to null images with early darkening of the blood pool
	Cardiac Sarcoidosis	Non-specific pattern: multiple foci of patchy LGE subepicardial, mid-wall or subendocardial distribution
**Secondary myocardial fibrosis**		
	Valvular heart disease	
	Mitral Valve Prolapse	LGE at the level of papillary muscles and inferolateral wall.
	Aortic stenosis	Patchy non-infarct LGE
	Coronary artery disease	Subendocardial or transmural LGE with coronary artery territory distribution
	Myocarditis	- Subepicardial, midwall, transmural scarring, often in the inferolateral wall. - Antero-septal mid-wall LGE pattern associated with worse prognosis
	Athlete's Heart	- “Benign” junctional spotty pattern. - “Stria” LGE pattern in postero-lateral wall associated with higher arrhythmic risk

*LGE, Late Gadolinium Enhancement; LV, left ventricular; RV, right ventricular*.

### Endomyocardial Biopsy: Strength and Limitations

Histopathological analysis of EMB represents the gold standard to confirm the presence of MF. Such procedure should be performed by an experienced operator, in a medical center with availability of expertise in cardiac pathology. In fact, EMB carries immediate and delayed risks for the patient such as perforation, arrhythmias, pneumothorax, vascular or nervous damage, bleeding or thrombosis (3, 5). In the study by Fowles and Mason on more than 4,000 biopsies, the overall complication rate was <1% and strictly dependent on the patient's clinical state (6). EMB can require venous or arterial access depending on the analyzed chamber. Usually, it is performed safely under a fluoroscopic or, less frequently, echocardiographic guidance. According to the latest guidelines, Class I Recommendation, Level of Evidence B, for EMB exists in the setting of: unexplained, new-onset heart failure of <2 weeks-duration associated with a normal-sized or dilated left ventricle in addition to hemodynamic compromise; unexplained new-onset heart failure of 2 weeks−3 months duration associated with a dilated left ventricle and new ventricular arrhythmias, Mobitz type II second- or third-degree atrioventricular (AV) heart block, or failure to respond to usual care within 1–2 weeks (5). However, only a small portion of the myocardium can be sampled and analyzed; therefore, sampling error may lead to missing the diseased myocardium. On the contrary, modern cardiac imaging techniques allow a non-invasive and comprehensive assessment of the entire myocardium, offering new potential in the study of MF and its clinical and prognostic value.

### Left Ventricular Fibrosis by Echocardiography

Information obtained by imaging methods, to be considered reliable markers of MF, should: correspond to histological specimens; correlate with known markers of MF (morphological and biochemical); track disease progression or regression after appropriate therapy; identify specific characteristics in patients affected by pathological conditions compared with controls. 2D echocardiography provides several systolic and diastolic function parameters but has not enough sensitivity and specificity for tissue characterization. STE has proven to derive indirect information about the presence of MF through the analysis of STE deformation parameters. Indeed, MF causes abnormal endocardial thickening by an increase in myocardial stiffness and consequent changes in cardiomyocyte mechanics, reflected in the deformation parameters assessed by STE (7, 8). In patients with advanced heart failure (HF) enlisted for heart transplantation (HTX) (8), global longitudinal strain (GLS) demonstrated greater accuracy for the prediction of MF (documented by Masson's staining on LV tissue samples obtained during HTX) compared with traditional indices of systolic function such as mitral annular plane systolic excursion (MAPSE), TDI-derived S', and ejection fraction (EF). The optimal cut-off point for GLS to predict severe MF was −10%. Also, global circumferential strain and apical rotation significantly correlated with the presence of MF (*r* = 0.61, *p* = 0.001 vs. *r* = 0.75, *p* = 0.0001, respectively). Collagen deposition may influence myocardial function based on its localization in different muscle layers too. Funabashi et al. (9) showed a greater reduction in GLS in subjects with HCM in which MF affected the endocardium compared to those with fibrotic lesions extended exclusively at the epicardium. Haland et al. (10) showed that the electromechanical dispersion, defined as the time delay between the beginning of the QRS and peak longitudinal strain in the 16 ventricular segments, correlates with fibrosis in CMR and is predictive of malignant ventricular arrhythmias. In patients with severe aortic stenosis, there is a correlation between MF and decrease in GLS, which confirmed a greater predictive value in detecting MF than global circumferential strain (11). Kansal et al. (12) also confirmed that a decrease in global circumferential strain occurred later than GLS in patients with MF of different etiologies. In patients with Fabry disease, a reduction in GLS at basal segments corresponded to fibrotic areas detected with CMR (13). In CAD, STE has been evaluated in different studies to analyze the response of the different ventricular components to subendocardial or transmural ischemia. A few years ago, in a rat model in which myocardial infarction was induced by ligation of the left anterior descending coronary artery, radial and circumferential strain at 4 weeks post-infarction were significantly decreased in the infarct area that included anterior and anterolateral wall and anterior septum. In those segments, the presence of MF was documented by histologic Masson staining with a significative correlation with strain values (*r* between −0.61 and −0.80, all *p* < 0.01) (14). In a population of 39 patients with a first anterior wall infarction with ST-elevation (15), treated with a primary percutaneous coronary intervention, the mean LS of the nine segments supplied by left anterior descending coronary artery was measured before discharge and compared to the infarct size 3 months after the acute event, assessed by CMR with LGE. Anterior wall LS had the strongest correlation (*r* = 0.68, *p* = 0.001); a GLS cut-off value of −11.5% in the segment supplied by left anterior descending was able to predict a large infarct size (that is at least 20% of the LV myocardium involved by the scar) with a sensitivity of 90% and a specificity of 73% (AUC 0.84). Despite these promising results, the use of STE is still limited by lack of standardization and specific cut-off values; moreover, it is strongly dependent on the echocardiographic image quality, which makes STE analysis challenging in the presence of poor acoustic windows, e.g., patients with lung emphysema, previous HTX, breast prostheses.

### Left Ventricular Fibrosis by Cardiac Magnetic Resonance

#### Late Gadolinium Enhancement

**Figure 3:**
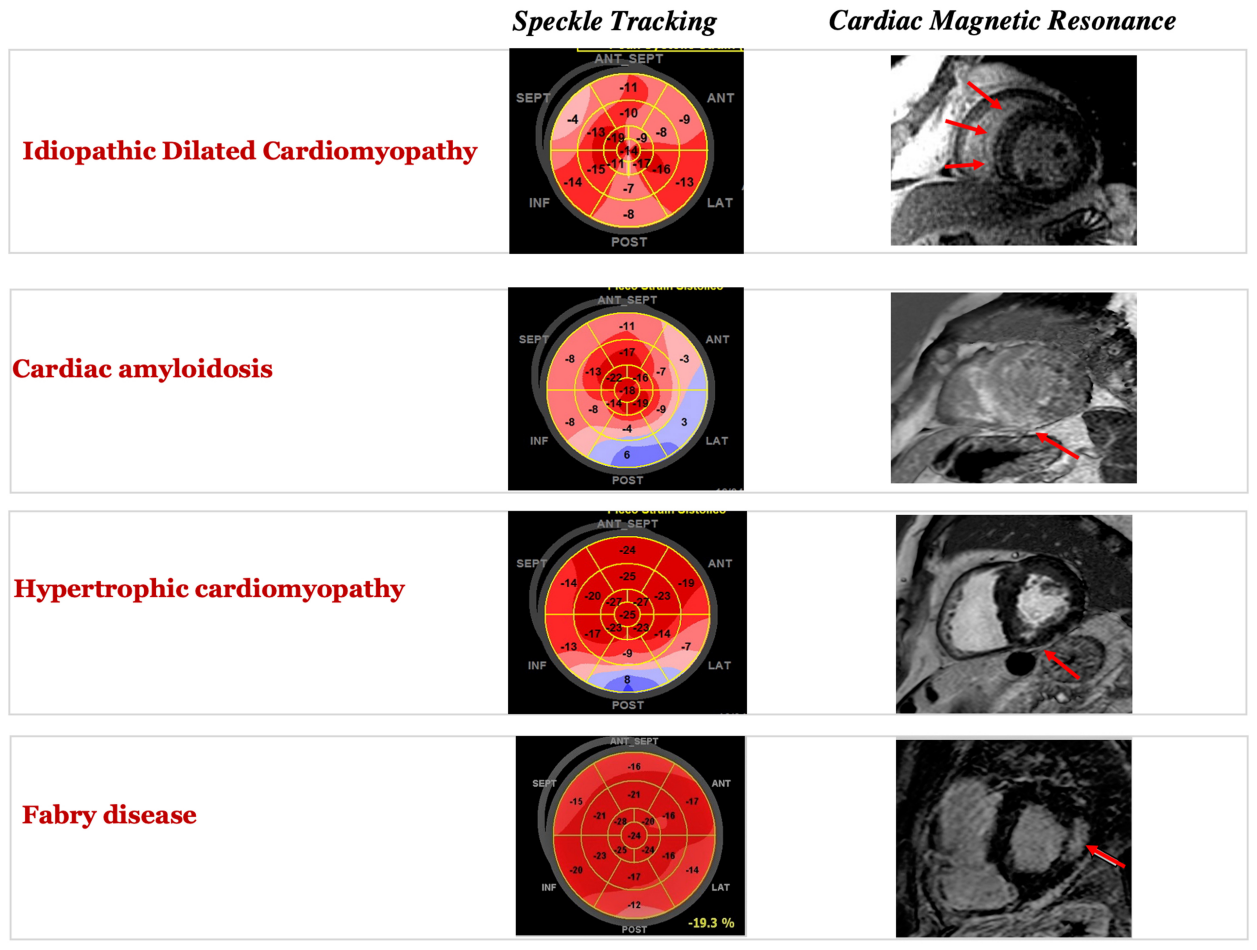
Typical patterns of myocardial fibrosis in different cardiomyopathies by speckle tracking bull's eye representation (left) and by cardiac magnetic resonance late gadolinium enhancement.

The introduction of LGE by CMR in the clinical arena has opened up the door to non-invasive myocardial tissue characterization. As opposed to STE, LGE allows a more direct “*in-vivo*” tissue characterization, highlighting areas with regional extracellular expansion, such as replacement fibrosis and scar. LGE module is acquired 10–20 min after paramagnetic contrast agent injection, by leveraging the different washout properties of normal and pathological myocardium. Gadolinium contrast agents accumulate in any expanded extracellular space and, due to their short T1 relaxation time, appear as a bright signal in inversion-recovery gradient-echo sequences, therefore outlining diseased areas from the normal myocardium. Over the last 20 years, the number of studies published on LGE has increased, highlighting this technique's diagnostic and prognostic power. Kim et al. initially demonstrated the relationship between the degree of LGE trans-murality and functional recovery after coronary revascularization, allowing myocardial viability assessment by CMR to risk-stratify patients and guide coronary revascularization (16). The presence of LGE and its quantification predicted adverse outcomes and mortality in coronary artery disease (17). While LGE was initially conceived to identify myocardial infarction areas, showing a close correlation to histopathology proven myocardial necrosis, it has later demonstrated to provide essential diagnostic information also in the non-ischemic clinical spectrum. Different LGE distribution patterns occur in the early stages of various non-ischemic pathological processes, thus guiding the cardiomyopathy phenotyping and diagnosis. For example, HCM usually presents patchy LGE in the hypertrophied segments and at a junctional level (18). In dilated cardiomyopathy, a frequent—although non-specific—sign is the so-called “mid-wall” stripe, an intramural region of fibrosis usually in basal and/or mid- septum (19). Myocarditis features subepicardial/transmural LGE, more often involving the inferolateral wall, but also other regions or with a more diffuse distribution (20). Anderson-Fabry disease presents with a characteristic mid-wall or subepicardial enhancement in the basal inferolateral wall (21). Amyloid deposits usually cause a diffuse subendocardial or transmural LV enhancement, frequent RV and biatrial involvement, with early darkening of the blood pool due to abnormal gadolinium kinetics (22). Figure 3 shows examples of typical patterns of MF assessed by STE and LGE across different cardiac diseases, and Table 1 summarizes main CMR findings. The presence of specific LGE patterns should always be interpreted in a comprehensive approach, taking into account info obtained from other CMR sequences (for example, myocardial oedema, highlighted with T2-weighted or T2 mapping sequences), as well as functional data from different cardiac imaging techniques such as STE. LGE is useful when regional areas of the myocardium are affected, but it lacks in evaluating diffuse fibrosis. Since the enhanced area is determined on the basis of the difference in signal intensity compared to the normal myocardium, in the presence of diffuse fibrosis, no differences will be observed. Techniques other than LGE are preferred when the entire myocardium is involved in the pathological process. Other LGE limitations (23) include the need for contrast agent injection and the difficulty in accurate absolute quantification of scar burden and gray zone areas presenting with intermediate signal intensity between normal and scarred tissue.

#### T1 Mapping and Extracellular Volume Quantification

New CMR sequences are emerging to better characterize and quantify diffuse interstitial MF. T1 mapping gives a numerical value (in milliseconds) for the T1 relaxation of the myocardium in a pixel-wise fashion and standardized scale. Parametric mapping techniques provide a better characterization of myocardial tissue composition both on the global and regional level, allowing the detection and quantification of diffuse histopathological changes—such as interstitial fibrosis—impossible to depict with LGE. By combining information from native and post-contrast T1 maps, the fraction of interstitial space can be measured as extracellular volume (ECV). Increased ECV might also result from interstitial edema or capillary expansions and must be taken into consideration and ruled-out when investigating diffuse MF. Even if T1 and ECV signals detected on imaging are non-specific and may be abnormal due to different pathophysiologic conditions, increased values of native T1 and ECV have shown good correlation with histologic evidence of interstitial MF (24, 25). ECV prognostic significance has been demonstrated in different disease subsets (26), such as amyloidosis (27), HF (28) and aortic stenosis (29), showing good reproducibility (30). Moreover, ECV demonstrated a robust association with clinical outcomes across the spectrum of ejection fraction and HF stages, adding incremental prognostic value in a large cohort of ischemic and non-ischemic cardiomyopathy (31). However, the application of T1 mapping in clinical practice is still limited due to lack of standardization of the acquisitions (different sequences available) (32) and the absence of standardized cut-off values between normal and pathological conditions. Many efforts have been made to achieve a better standardization, with two consensus statements published in 2015 and 2017 (33, 34) and commercial sequences getting available. Ongoing large registries will provide further insights into this research area.

#### Feature Tracking CMR and CMR Tagging

Another CMR application gaining popularity in the (indirect) assessment of diffuse MF is feature-tracking CMR (FT-CMR), allowing the evaluation and quantification of myocardial deformation before any identifiable changes in ejection fraction. Research studies have shown a correlation between LGE and strain assessed by FT-CMR in non-ischemic cardiomyopathies (35, 36), as well as its prognostic implications in different conditions (37). Similarly to STE, FT-CMR investigates the functional consequences of MF, analyzing the active myocardial deformation (strain) in three orthogonal directions: radial, circumferential and longitudinal. FT-CMR is based on the post-processing of standard steady-state free precession (SSFP) cine images. The endo- and epicardial myocardium borders are usually manually traced in the end-diastolic phase, then automated algorithms allow the tracking of distinctive anatomical “features,” typically identified along the blood cavity-myocardial interface, and follow them along the cardiac cycle (38). Global and segmental LV strain, strain-rates, and LV rotational mechanics can be obtained from standard long- and short-axis SSFP views. This technique is increasingly used, thanks to the easy and fast post-processing without the need for dedicated acquisition. However, it has to be reminded that FT-CMR has substantially lower spatial and temporal resolution compared to STE. Global (rather than segmental) strain values appear the most reproducible (39, 40). Strain analysis by CMR can also be performed through CMR tagging, less widely diffused in clinical practice, but extensively validated *in vitro* and *in vivo* (41, 42). CMR tagging allows a more direct assessment of myocardial deformation by measuring physical properties of the tissue. This technique is based on perturbations of the myocardial tissue magnetization, resulting in dark lines, forming a grid of tissue markers known as tags. Compared to FT-CMR, CMR tagging provides more reproducible measurements, since the tags are more clearly defined and easily tracked than the natural features (39). However, time-consuming post-processing using dedicated software solutions is needed. For these reasons, CMR tagging has mainly remained a research tool.

### Imaging Left Ventricular Fibrosis by Computed Tomography

Emerging data suggest that computed tomography (CT) can be complementary to echocardiography and CMR, providing both anatomical and functional analysis of LV but also tissue characterization (43). Its main advantages are excellent spatial resolution and high temporal resolution up to 66 milliseconds with short acquisition time. It is worth noting that CT signals are related to X-ray beam attenuation by iodine molecules, thus representing more scar-specific imaging (44). Delayed phase cardiac CT, which means that scanning is performed 5–15 min following coronary CT angiography, can assess MF and scarring. Regions of fibrosis reduce the washout of iodinated contrast medium, which can be then visualized on delayed phases (45). CT, as well as CMR, using an extravascular, extracellular contrast agent, allows the quantification of ECV, and in turn myocardial fibrosis (46). A significant correlation between myocardial ECV quantification determined by CMR and cardiac CT was demonstrated (47) as well as a relevant correlation between CT-derived myocardial ECV and percentage of histological fibrosis (48). Some studies have also confirmed that infarct size can be quantified using delayed phase CT with a good correspondence with CMR. Patterns of delayed enhancement (DE) in different conditions are similar to CMR: for example, cardiac sarcoidosis can show subepicardial, transmural or mid myocardial DE with an increase of ECV; in cardiac amyloidosis, DE can be circumferential subendocardial or transmural; in dilated cardiomyopathy there is a linear mid-wall DE. CT can help differentiate myocarditis from acute coronary syndrome by detecting mid-wall or subepicardial DE and concomitant absence of significant CAD (49, 50). Overall, as suggested by Takaoka et al. (51) even if CT shows an inferior inter-observer agreement compared to CMR, it should be considered for the detection of DE in patients with wall motion abnormalities, in particular, if contrast detects are observed in LVM in the early phase then late-phase acquisition should be performed. In terms of more novel methodology, dual-energy CT, which uses double energy acquisition and is performed without the use of contrast, has been demonstrated to perform better than single energy approach and is more comparable to CMR in LGE and ECV quantification (52). There is an increasing interest into CT feature tracking as an alternative measure of the global myocardial strain of the LV. Recent studies show promising results with a good agreement compared to FT- CMR (53).

### Clinical Applications: Arrhythmias and Sudden Cardiac Death

**Figure 4:**
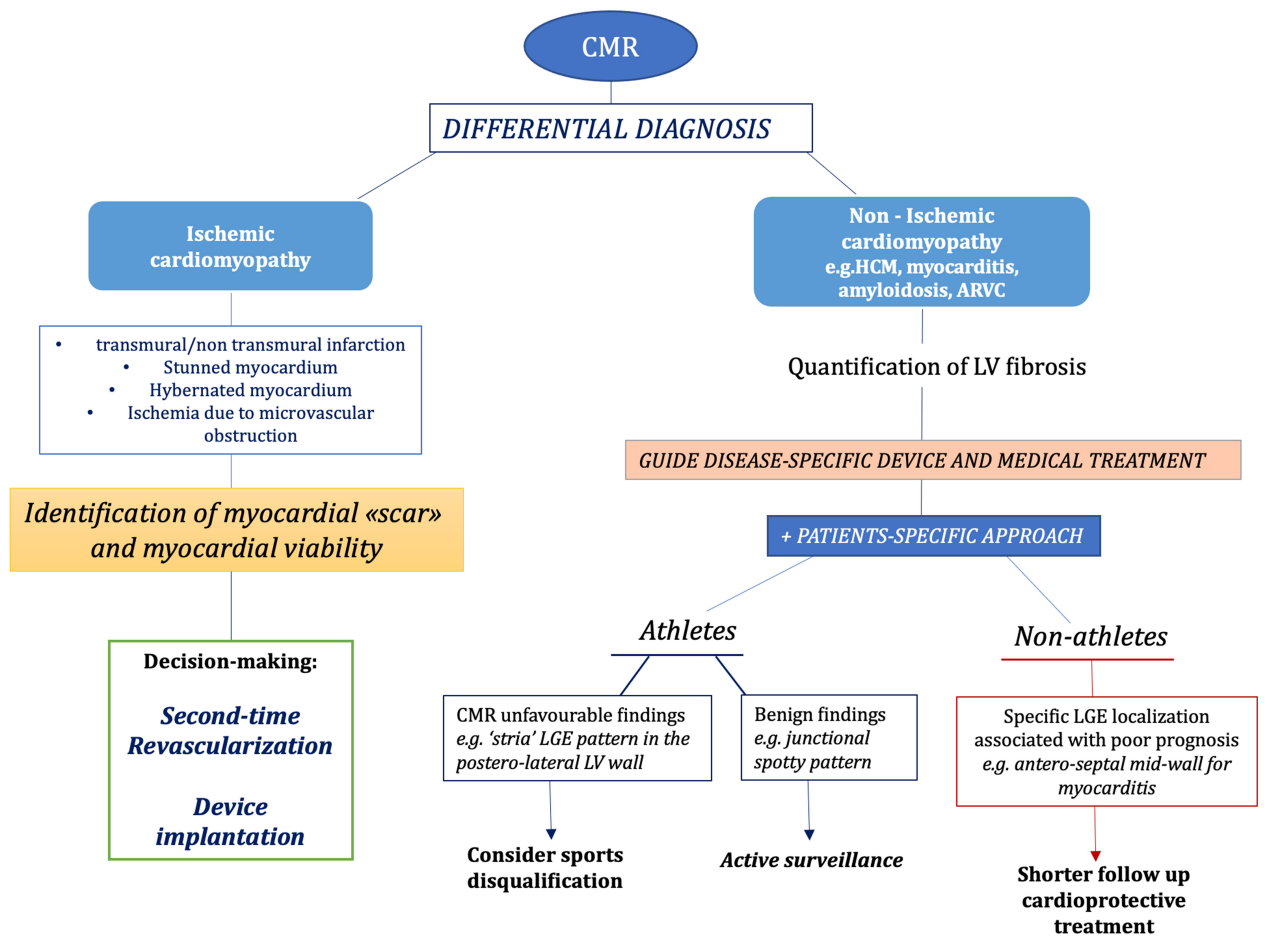
Proposed diagnostic algorithm for the role of different imaging modalities e.g., cardiac magnetic resonance (CMR) in the diagnosis of disease causing left ventricular myocardial fibrosis with increased risk of sudden cardiac death. ARVD, arrhythmogenic right ventricular cardiomyopathy; CMR, cardiac magnetic resonance; HCM, hypertrophic cardiomyopathy; LGE, late gadolinium enhancement; LV, left ventricular.

Sudden cardiac death (SCD) is responsible for 25% of 17 million cardiovascular death every year in the World, and the great majority of these deaths have an arrhythmic origin. The underlying causes vary according to age, with channelopathies and cardiomyopathies prevailing in young people, while degenerative diseases and ischemic heart disease being more common in older people. In order to prevent these events, risk stratification is crucial but still very challenging. Ventricular MF is a critical substrate for the genesis of ventricular arrhythmias (VAs): indeed, within fibrotic tissue, the slow and heterogeneous conduction favors re-entrant circuits, increasing vulnerability to ventricular tachycardia and ventricular fibrillation (54, 55). Therefore, the identification and quantification of scarred myocardial areas appear crucial. The presence of ventricular fibrosis could be a strong predictor of VAs and SCD in several cardiac diseases (56, 57), suggesting the great potential of MF as risk stratification marker (56). Indeed, accumulating evidence on ventricular fibrosis is available in patients with HCM, ischemic cardiomyopathy (ICM), and non-ischemic cardiomyopathies (NICM). In patients with ICM, ventricular fibrosis assessed by LGE is a powerful predictor of VAs (56), and the progressive extension of the infarct gray zone is a significant predictor of appropriate ICD therapy (58). LGE ability to predict VAs was confirmed in both ICM and NICM populations in two large meta-analyses (56, 59), where VAs resulted more common in patients with larger LGE extent compared with patients with negative LGE (annualized event rate 8.6 vs. 1.7%; *p* < 0.0001). Notably, LGE correlated with arrhythmic events in both etiology- and EF-based subgroups. Several studies demonstrated the predictive power of LGE for SCD in NICM patients (60). Gulati et al. (61) showed that the presence of mid-wall fibrosis was correlated with the occurrence of VA in NICM patients with an adjusted hazard ratio of 4.61 (95% CI: 2.75–7.74). Similarly, Kuruvilla et al. (62) demonstrated that NICM patients with fibrosis had an annualized risk for arrhythmic events of 6 vs. 1.2% of patients without fibrosis (*p* < 0.001). Notably, LGE was an independent predictor of monomorphic VT but not polymorphic VT or VF, likely because of the limited capability of LGE to detect the diffuse fibrotic substrate of more complex VT. The diagnostic and prognostic value of fibrosis is relevant also in HCM. Indeed, the LGE assessment is commonly used in the workup of cardiomyopathies and is very useful to differentiate distinctive forms of cardiac hypertrophy, including amyloidosis, HCM, and athlete's heart (63). Although data on LGE prognostic value in HCM patients are less uniform than in ICM/NICM patients, recent evidence supports the LGE predictive power for SCD, with presence and extent of LGE appearing strong independent predictors of SCD (64), whether assessed by semi-quantitative or quantitative methods (65). Chan et al. (66) also demonstrated that the incidence of SCD in HCM was directly proportional to the percentage extent of LGE, increasing from 1 per 1,000 person-year in the absence of LGE to 10 at LGE ≤ 10%, 18 at 11–19%, and 24 at LGE 20% (*p* = 0.001 for trend). Chiribiri et al. (67) showed that HCM patients had abnormal rest perfusion associated with the presence and distribution of myocardial scar and supporting the assessment of rest perfusion abnormalities to identify patients with an increased incidence of non-sustained VT. Interestingly, in 43 young patients (<40 years) with mitral valve prolapse who died from SCD, the presence of MF was demonstrated at histology at the level of papillary muscles in all subjects and of LV inferolateral wall in 83% (40). LGE was present in 93% of patients with the same regional distribution, showing the potential role of CMR in this neglected cause of SCD (68). In a population of young athletes, a “stria” LGE pattern in the postero-lateral LV wall has been associated with a higher arrhythmic risk compared to the “benign” junctional spotty pattern (69), and non-ischemic LV scar has been identified as a relevant cause of sports-related SCD (70). As a consequence, the presence of a non-ischemic LV scar should be suspected on the basis of abnormal resting ECG and presence of uncommon arrhythmias in these subjects (71–73), leading to active surveillance and possibly sports disqualification (74). In the ITAMY (Italian multicenter study on Acute Myocarditis)—overall enrolling a population of 386 patients with acute myocarditis with preserved EF—Aquaro et al. observed that an anteroseptal mid-wall LGE pattern was associated with a worse prognosis than other patterns of presentation (75). In conclusion, the non-invasive assessment of myocardial fibrosis is revolutionizing the diagnostic, prognostic, and therapeutic approach of patients with cardiomyopathies and arrhythmias. The evaluation of presence, extent, and progression of ventricular fibrosis is a promising tool for the management of patients at risk of SCD and, given the suboptimal risk prediction of LVEF, further research is needed to confirm the role of ventricular fibrosis to guide clinical decision making and to improve SCD risk stratification algorithms. Figure 4 shows a proposed possible algorithm to improve SCD management by CMR.

## Fibrosis and Left Atrium

### Main Causes of Left Atrial Fibrosis and Its Identification by Echocardiography

Because of its peculiar anatomy and thin walls, LA is extremely sensitive to internal and external insults. In the presence of chronic pressure overload, like hypertension or aortic stenosis (76), the diastolic dysfunction and increase in LV filling pressures are responsible for LA maladaptive remodeling and fibrosis, causing LA enlargement and decreased distensibility. Diastolic function abnormalities lead to LA remodeling also in conditions of volume overload, such as mitral regurgitation, or both pressure and volume overload, such as heart failure (8). LA MF is associated with myocyte loss, either by apoptosis or necrosis and has many pathological ways in common with LV fibrosis. The presence of LA fibrosis correlated with worse outcome in different cohorts, particularly for the higher arrhythmic burden (77) being a substrate for AF or other supraventricular arrhythmias. Its early identification with non-invasive techniques would be useful to identify those patients with higher extent of “electrical” remodeling who would benefit from more aggressive therapies to prevent AF onset and recurrence (78).

#### Speckle Tracking Echocardiography

Several studies demonstrated how a reduction of LA function assessed by STE predicts the presence of LA fibrosis. Overcoming the majority of the limitations of standard echocardiographic measures such as atrial EF, transmitral flow, and TDI analysis, the application of STE to characterize atrial function is increasing, allowing deriving measures of reservoir and contractile function. The most used index is peak atrial longitudinal strain (PALS), an excellent marker of atrial reservoir function, which concurs with its maximum distension. Normal PALS cut-off values are 42.2% ± 6.1 (79). The presence of fibrosis in LA walls produces a relevant decrease of PALS anticipating atrial dilation, as a sign of reduced compliance of the atrium (80, 81). Kuppahally et al. (82) evaluated 65 patients with paroxysmal (44%) or persistent (56%) atrial fibrillation (AF) by both CMR with LGE and STE. An inverse correlation between PALS and the presence of MF (*r* = −0.50, *p* = 0.003) was demonstrated. MF in patients with mitral regurgitation has been investigated by studies using STE and histopathological analysis (83, 84). In 28 patients undergoing cardiac surgery for chronic mitral regurgitation secondary to mitral valve prolapse, a close negative correlation was found between global PALS, and the degree of LA MF on anatomical samples by Masson's trichrome staining (*r* = −0.82, *p* < 0.0001). Conversely, LA volume, LA emptying fraction and E/e' ratio poorly correlated with the presence of MF (83). The main traditional concerns about the clinical use of LA strain are the lack of a dedicated software and its challenging execution, due to possible atrial foreshortening or inadequate tracking in areas of pulmonary veins outflow. However, the recent standardization and “how-to” papers of the European Association of Cardiovascular Imaging (EACVI) (85, 86), as well as the development of specific LA-strain softwares, have paved the way toward a standardized use of LA strain and easier training even for non-expert operators, reaching high feasibility results (79).

### Assessing Left Atrial Fibrosis by CMR and Clinical Applications: Atrial Fibrillation

Technical improvements have prompted the use of LGE CMR for the possible identification and quantification of LA fibrosis too. The assessment of LA fibrosis is challenging due to limitations in CMR image resolution, thin atrial walls (1–2 mm) and highly variable atrial shape. Data on LA fibrosis quantification by LGE CMR suggest that its presence may predict the recurrence of AF following catheter ablation (87, 88). Pre-procedural LA fibrosis assessment by LGE CMR guides patient selection and strategy planning (89), while post-procedural assessment can identify ablation scarring and line gaps (90). Daccarett et al. (91) identified four categories of LA remodeling (Utah stages I–IV) based on the amount of LA fibrosis by LGE-CMR and showed that the extent of LA remodeling correlates with CHADS2 score and stroke risk. However, CMR techniques for the quantification of LA fibrosis reported in the literature are heterogeneous and there is a lack of normative and reproducibility data. These limitations can potentially limit its extensive application in clinical practice (92). The presence of atrial fibrosis could guide an effective ablation by the identification of atrial regions that are already widely remodeled by fibrotic tissue; furthermore, it can predict the recurrence of AF (93, 94) and improve the selection of candidates to transcatheter ablation or electrical cardioversion. When PALS is severely impaired, the MF could be at an advanced stage and the procedure has a high probability to be ineffective (95, 96). Parwani et al. (96) performed LA strain analysis before the first catheter ablation (CA) in 102 patients with persistent AF and correlated PALS with recurrence of arrhythmia during a mean follow up of 15 months, with or without antiarrhythmic drugs (primary endpoint). The 55 subjects who relapsed AF had significantly reduced LA strain (9.7 ± 2.4% vs. 16.2 ± 3.0%, *p* < 0.001) and the cut-off value of 10% defined the higher risk of recurrence. PALS was the strongest predictor of recurrence of AF (HR 2.4–16.9, *p* < 0.001) and this was confirmed also after the second CA. Similar data have been published also using CMR (88, 97). An emerging CMR application in the evaluation of LA fibrosis is T1 mapping. Beinart et al. found post-contrast LA T1 times to be shorter in patients with AF compared to healthy subjects (98). Post-contrast LA T1 mapping with values < 230 ms was associated with a higher risk of AF recurrence (99). In contrast, native (pre-contrast) atrial T1 mapping showed increased values in patients with recurrence of AF, independently predicting poor outcome following ablation therapy. Moreover, a significant correlation between LA enhancement on LGE CMR and LA T1 relaxation times suggested that both parameters can detect and quantify LA wall fibrosis (100). Because of the limited spatial resolution of current T1 mapping techniques and the thin LA wall, atrial T1 mapping is still in the experimental phase. Higher resolution CMR tehniques in the future may help facilitate a more detailed and comprehensive atrial tissue characterization and prompt its use also in clinical practice. FT-CMR has recently been extended to the assessment of global longitudinal LA strain and strain rate analysis. The close correlation of impaired LA function and MF as determined by LGE and T1-mapping has been demonstrated (101).

**Figure 5:**
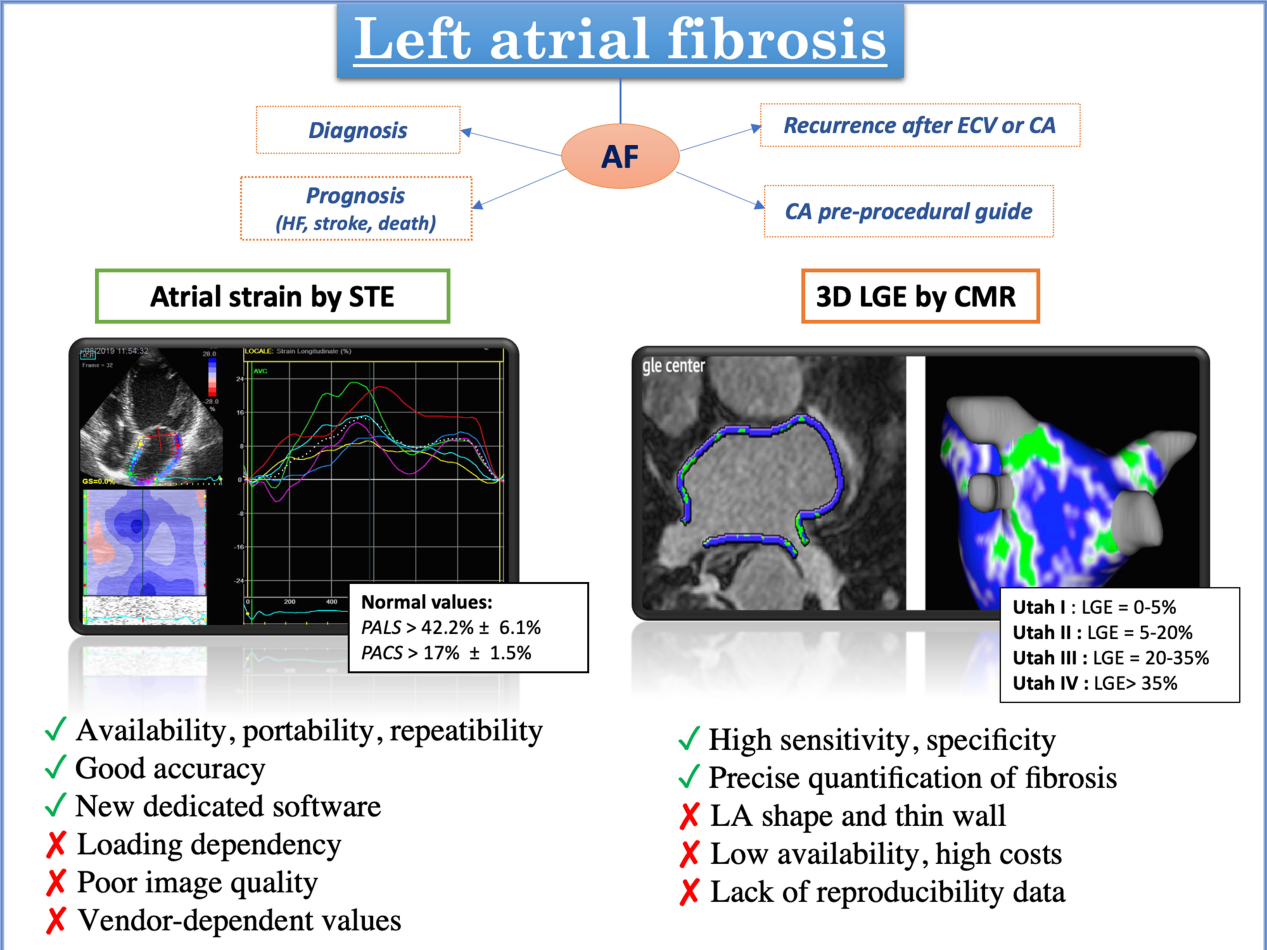
Different features and applications of advanced imaging modalities for the evaluation of left atrial fibrosis: on the left, evaluation of left atrial deformation by speckle tracking echocardiography; on the right, left atrial 3D model rendering by cardiac magnetic resonance obtained tracing the left atrial wall and quantifying late gadolinium enhancement signal with different color coding (blue: normal tissue; green: fibrosis) [adapted from Siebermair et al. (97)].

**Figure 6:**
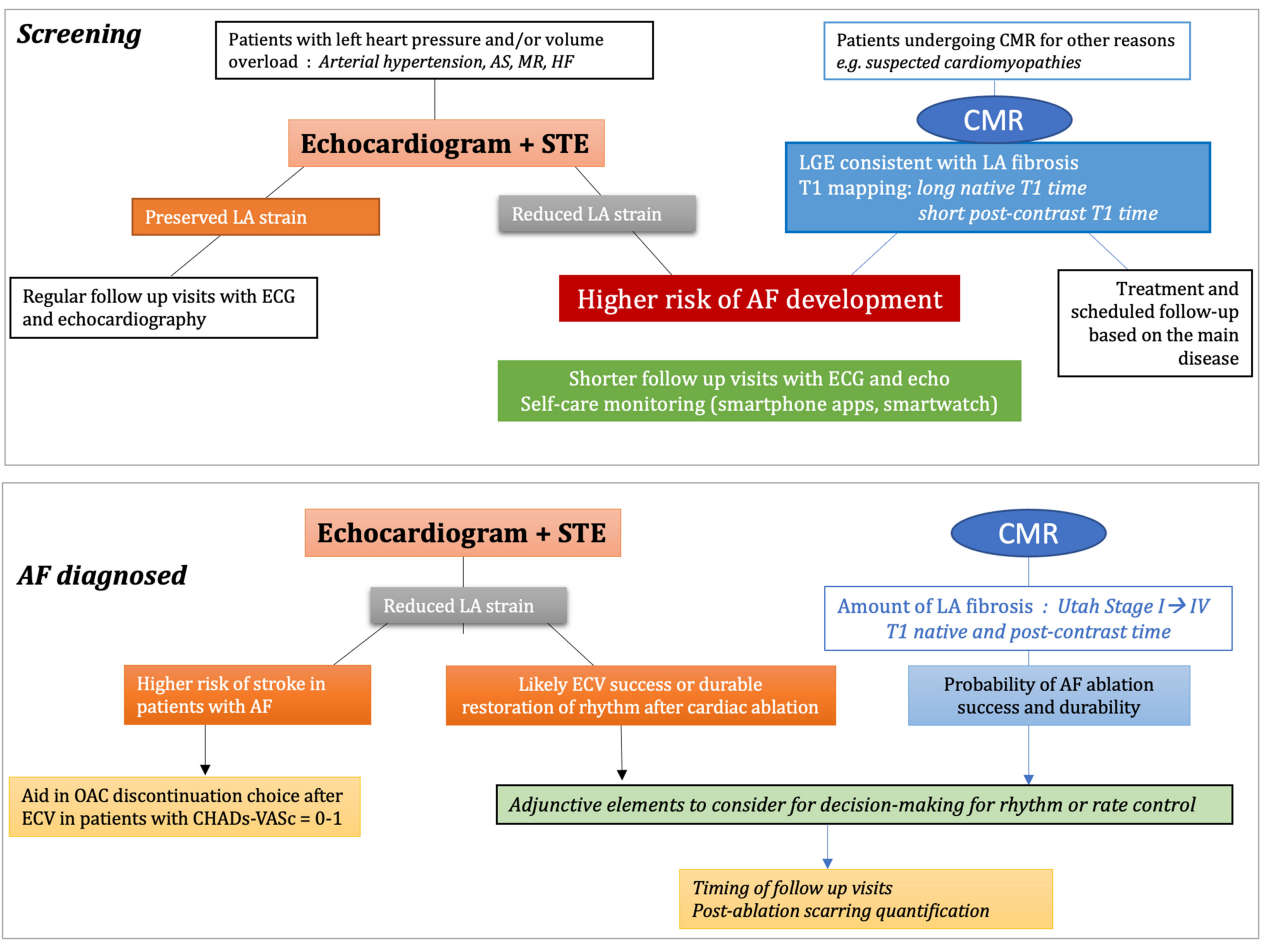
Proposed algorithms integrating speckle tracking echocardiography (STE) and cardiac magnetic resonance (CMR) for the screening and management procedures of atrial fibrillation. AF, atrial fibrillation; AS, aortic stenosis; ECG, electrocardiogram; ECV, extracellular volume; HF, heart failure; LA, left atrial; MR, mitral regurgitation; OAC, oral anticoagulants.

Figure 5 resumes the advantages/disadvantages and clinical applications of STE and CMR for the assessment of LA fibrosis, Figure 6 shows possible algorithms for the management of patients with suspected or known AF, by STE and CMR.

## Imaging Left Heart Fibrosis: Current Limitations and Future Perspectives

MF has been recognized as a common underlying factor for several clinical settings since the early 1930s (102). Since then, non-invasive identification of this morphological myocardial abnormality has been one of the holy grails of researchers in cardiology. The earliest clinical studies tried to correlate electrocardiographic changes with MF. Slurring, notching, decreased voltages, abnormal S-T-T configurations, and widened QRS intervals were found to be supportive of MF development (103). The introduction of ultrasound enhanced the likelihood of identifying fibrosis non-invasively and set a new goal: the possibility of direct visualization of the fibrotic wall, sometimes seen as hyper-refringent on ultrasound, as well as its indirect consequences on myocardial contraction and relaxation. Needless to say, the suboptimal sensitivity of the above-mentioned approaches was not sufficient to include fibrosis to the clinical decision-making process. However, the recent adoption of the STE technique has taken on the challenge of overcoming the sensitivity and reproducibility limitations of conventional ultrasound analysis. The knowledge on the pathophysiology of cardiac fibrosis has further advanced with the advent of LGE CMR and its direct and reliable identification and quantification of focal MF: the literature is currently flourishing with studies on the genesis, progression, potential regression and treatment of LV fibrosis as assessed by LGE CMR. CMR mapping techniques appear promising in investigating diffuse interstitial fibrosis, but more data are needed before their introduction into clinical practice. Even if many Authors have widely demonstrated the value of these two imaging modalities, the lack of disease-specific cut-off values for reference still limit their introduction in the decision-making algorithms of everyday clinical practice. Therefore, hopefully future studies should be focused on the research of reliable cut-off values of STE and CMR parameters to quantify MF for diagnostic and prognostic purposes in the different diseases. Equally, a more extensive use of cardiac CT with definition of cut-offs, would improve MF study. The detection of MF has been an area of extensive research beyond the field of cardiac imaging. Indeed, multiple biomarker approaches, mainly based on endogenous non-coding RNA molecules called micro-RNA, are currently under consideration (104), and they could complement or eventually challenge the current cardiac imaging techniques in the future.

## Conclusions

The continuous evolution and rapid advances of non-invasive multimodality cardiovascular imaging sets the stage for detecting myocardial fibrosis as a promising approach to improved clinical management of cardiovascular disease. Early and accurate detection of structural abnormalities of the myocardium has large potential to favorably influence the course of cardiac pathologies by increasing the efficacy of specific treatments and slowing disease progression, eventually leading to improved long-term cardiovascular outcome. Randomized research is eagerly awaited to test the effectiveness of imaging biomarkers in the management of multiple cardiovascular conditions.

## Author Contributions

GEM, FD'A, SM, and MC contributed to the conception of the review. GEM, FD'A, GV, and GB performed the literature search and data analysis. LC gave a substantial contribution to the requested revisions of the manuscript. All authors contributed to this manuscript draft and validation, critically revised the work, and approved the final manuscript.

## Conflict of Interest

The authors declare that the research was conducted in the absence of any commercial or financial relationships that could be construed as a potential conflict of interest.
